# Enhancing Omicron Sublineage Neutralization: Insights From Bivalent and Monovalent COVID‐19 Booster Vaccines and Recent SARS‐CoV‐2 Omicron Variant Infections

**DOI:** 10.1111/irv.70000

**Published:** 2024-10-08

**Authors:** Hye Won Jeong, Rare Rollon, Se‐Mi Kim, Juryeon Gil, Mark Anthony Casel, Hyunwoo Jang, Jeong Ho Choi, Seung‐Gyu Jang, Josea Carmel Lazarte, Hee‐Sung Kim, Jun Hyoung Kim, Young Ki Choi

**Affiliations:** ^1^ College of Medicine and Medical Research Institute Chungbuk National University Cheongju Republic of Korea; ^2^ Department of Internal Medicine Chungbuk National University Hospital Cheongju Republic of Korea; ^3^ Center for Study of Emerging and Re‐emerging Viruses Korea Virus Research Institute, Institute for Basic Science (IBS) Daejeon Republic of Korea

**Keywords:** antibody responses, bivalent, monovalent, omicron sublineages

## Abstract

**Background:**

Omicron variants have rapidly diversified into sublineages with mutations that enhance immune evasion, posing challenges for vaccination and antibody responses. This study aimed to compare serum cross‐neutralizing antibody responses against various SARS‐CoV‐2 Omicron sublineages (BA.1, BA.5, XBB.1.17.1, FK.1.1, and JN.1) in recipients of monovalent COVID‐19 boosters, bivalent booster recipients, and individuals who had recovered from Omicron BA.5 infections.

**Methods:**

We conducted a micro‐neutralization assay on serum samples from monovalent BNT162b2 booster recipients (*N* = 54), bivalent BNT162b2 booster recipients (*N* = 24), and SARS‐CoV‐2 Omicron BA.5‐recovered individuals (*N* = 13). The history of SARS‐CoV‐2 Omicron infection was assessed using ELISA against the SARS‐CoV‐2 NP protein.

**Results:**

Bivalent booster recipients exhibited significantly enhanced neutralization efficacy against Omicron sublineages compared to those who had received monovalent booster vaccinations. Omicron BA.5‐recovered individuals displayed similar neutralizing antibodies (NAbs) to the bivalent booster recipients. Despite the improved neutralization in bivalent recipients and BA.5‐recovered individuals, there were limitations in neutralization against the recently emerged Omicron subvariants: XBB.1.17.1 FK.1.1, and JN.1. In both monovalent and bivalent booster recipients, a history of Omicron breakthrough infection was associated with relatively higher geometric mean titers of NAbs against Omicron BA.1, BA.5, and XBB.1.17.1 variants.

**Conclusion:**

This study underscores the intricate interplay between vaccination strategies, immune imprinting, and the dynamic landscape of SARS‐CoV‐2 variants. Although bivalent boosters enhance neutralization, addressing the challenge of emerging sublineages like XBB.1.17.1, FK.1.1, and JN.1 may necessitate the development of tailored vaccines, underscoring the need for ongoing adaptation to effectively combat this highly mutable virus.

## 1 Introduction

The emergence of the Omicron variant (B.1.1.529) of severe acute respiratory syndrome coronavirus 2 (SARS‐CoV‐2) on November 24, 2021, marked a significant turning point in the ongoing COVID‐19 pandemic, and it was designated as a variant of concern (VOC) by the World Health Organization (WHO) just 2 days later. Unique from the other VOCs, Omicron exhibits a multitude of mutations in its spike protein (S), which is primarily responsible for the virus's interaction with host cells, and these mutations may contribute to Omicron's marked transmissibility [1, 2]. Since its initial emergence, the Omicron variant has given rise to various sublineages (BA.1, BA.2, and BA.4/BA.5), each characterized by distinct genetic and antigenic features from the parental Omicron lineages and with increased transmissibility and ability to evade neutralizing antibodies (NAbs) induced by previous infections or vaccinations [3, 4, 5].

The Omicron XBB sublineage, which accounted for over 90% of circulating variants in late 2022, exhibited heightened prevalence in most countries, except in Australia and New Zealand [6, 7, 8]. Among these sublineages, XBB.1.17.1 stands out as an XBB.1‐like sublineage, originating from a recombination event between two BA.2 lineages, BJ.1 and BA.2.75, which appeared to be dominant in Central/West Africa between January and April of 2023. XBB.1.17.1 was also found in France (22.0%), the United States (12.0%), the United Kingdom (11.0%), Spain (8.0%), and Sweden (7.0%) [9, 10]. In parallel, FK.1.1, derived from a non‐XBB branch of the Omicron variant (CH1.1), is prevalent, particularly in the United Kingdom, where it accounts for approximately 30% of cases. FK.1.1 is characterized by additional mutations in the spike gene, including D215G and Q613H, and exhibits varying prevalence in other countries including Australia (29.0%), New Zealand (32.0%), South Korea (14.0%), the United States (11.0%), and Japan (6.0%) [9]. The Omicron JN.1 sublineage, a descendant of the BA.2.86 variant, emerged in September 2023 and become predominant worldwide from December 2023 to January 2024. This specific sublineage was noted for its enhanced immune evasion ability, attributed to its decreased affinity for ACE2, which affected the efficacy of Class 1 NAbs [11].

The emergence of these diverse Omicron sublineages, each harboring distinct spike protein mutations, has raised concerns regarding the reduced efficacy of current vaccines against these variants, particularly when compared to the initial BA.4/BA.5 lineages [10, 11, 12]. In response to these concerns, bivalent SARS‐CoV‐2 vaccines, which combine the original vaccine and an Omicron‐specific component (BA.4/BA.5), have been deployed globally. However, comprehensive evaluations comparing the efficacy of bivalent vaccines with that of monovalent vaccines (based on ancestral strain) against recently circulating Omicron XBB and JN.1 subvariant viruses remain limited. Growing evidence of antibody evasion by these emerging sublineages [11, 12, 13, 14] underscores the need for continuous surveillance of the immunological landscape across communities to foresee the future COVID‐19 burden. In light of this, our study aims to assess the cross‐neutralizing titers and antibody persistence in the sera of monovalent BNT162b2 booster (based on ancestral strain) recipients, bivalent BNT162b2 booster (based on ancestral and Omicron BA.4/BA.5) recipients, and those post SARS‐CoV‐2 Omicron BA.5 infection with no recent booster vaccination.

## 2 Materials and Methods

### 2.1 Patients and Specimens

We enrolled healthcare workers (HCWs) with varying vaccination statuses for this study. We recruited individuals who received a monovalent BNT162b2 vaccine (30 μg mRNA targeting the ancestral SARS‐CoV‐2 strain) as their booster dose and others who received a bivalent BNT162b2 vaccine (comprising 15 μg of mRNA targeting the ancestral SARS‐CoV‐2 strain and 15 μg of mRNA directed against the BA.4/BA.5 variants) as their booster shot between August 2022 and January 2023. All subjects had three‐ or four‐dose monovalent BNT162b2 vaccinations before study enrollment. We also recruited 13 HCWs who had recently experienced SARS‐CoV‐2 Omicron BA.5 infection and had received only three doses of monovalent BNT162b2 vaccine. As the SARS‐CoV‐2 infection in these subjects was confirmed during a period when national SARS‐CoV‐2 variant surveillance in South Korea reported over 95% of isolates were of the BA.5 sublineage (September 2022) [15], the subtype of their causative SARS‐CoV‐2 was considered to be the BA.5 sublineage. Serum samples were collected at 1, 3, and 6 months following their last booster vaccination or diagnosis of BA.5 sublineage infection and prepared for subsequent micro‐neutralization assays and enzyme‐linked immunosorbent assays (ELISA). At study enrollment, monovalent or bivalent booster recipients were surveyed regarding previous SARS‐CoV‐2 infections using a questionnaire, and infection was confirmed using an ELISA against the NP protein of SARS‐CoV‐2. HCWs who reported no previous SARS‐CoV‐2 infection and tested negative in the ELISA against SARS‐CoV‐2 NP protein were categorized as having no prior SARS‐CoV‐2 infection (Figure S1A).

### 2.2 ELISA Against SARS‐CoV‐2 NP Protein

For total antibody detection, we adapted an ELISA method based on a previously described protocol [16]. Briefly, flat‐bottom Immuno plates (SPL Life Sciences) were coated with SARS‐CoV‐2‐NP protein (1 μg/mL) in a bicarbonate–carbonate buffer and incubated overnight at 4°C. Subsequently, plates were washed three times with PBS containing 0.05% Tween 20 (PBST) followed by the addition of 150 μL blocking buffer (7.5% skim milk) per well. Plates were incubated for 1 h at 37°C and then washed three times with PBST.

Collected sera were prepared at a 1:200 dilution in 5% skim milk and added at 100 μL per well. The plates were incubated for 3 h at 37°C and then washed five times with PBST. A secondary antibody, anti‐human IgH + L polyclonal antibody‐HRP conjugate, was added at a dilution of 1:10,000 in PBST, followed by a 1‐hour incubation at 37°C. The plates were washed seven times with PBST. Finally, 50 μL of 3,3′,5,5′‐tetramethylbenzidine (TMB) (SeraCare, Milford, MA, USA) was added for 10 min for color development, and the reaction was halted by adding 50 μL of 1 N sulfuric acid (H_2_SO_4_). Optical density at 450 nm (OD450) was measured using the iMark Microplate Absorbance Reader (Bio‐Rad, USA).

### 2.3 Micro‐neutralization Assay Against SARS‐CoV‐2 Sublineages

NAbs against various SARS‐CoV‐2 strains, including the ancestral strain, as well as BA.1, BA.5, XBB.1.17.1, F.K.1.1, and JN.1 Omicron variants, were assessed using the collected serum samples. The micro‐neutralization assay was performed on serum specimens using Vero E6 cells (ATCC#: Vero C1008). Serum specimens were first inactivated at 56°C for 30 min. Subsequently, 50 μL of a twofold serially diluted serum sample (starting from 1:10) was mixed with 50 μL of 100 median tissue culture infectious dose (TCID_50_) of each SARS‐CoV‐2 strain. These mixtures were then incubated at 37°C for 1 h to neutralize the infectious virus and subsequently transferred to Vero E6 cell monolayers. The cells were incubated at 37°C with 5% CO_2_ for 1 h, followed by a media change. Gentian violet staining (1%) was employed to stain and fix the cell culture layer. The neutralizing dilution of each serum sample was determined by identifying the well with the highest dilution causing at least a 50% reduction in cytopathic effect (CPE), which was considered the NAb titer. A dilution equal to 1:10 or higher was considered neutralizing. In cases where the NAb titer was not detected, it was recorded as 5.

## 3 Results

A total of 54 monovalent booster (based on ancestral strain) recipients (all of whom had previously received 3 doses of ancestral vaccine), 24 bivalent booster recipients (based on ancestral and BA.4/BA.5 variants) including 13 individuals with previous 3 doses of ancestral vaccine and 11 individuals with previous 4 doses of ancestral vaccine, and 13 HCWs who had recovered from SARS‐CoV‐2 Omicron BA.5 variant infections were included in this study. Based on the responses to our questionnaire, all previously reported SARS‐CoV‐2 infections among the monovalent and bivalent booster recipients could be attributed to the Omicron variant, as determined by the date of diagnosis. The demographic information for enrolled subjects is summarized in Table 1.

**TABLE 1:** Demographic information including age and sex, previous SARS‐CoV‐2 infection, and previous vaccination history of enrolled individuals in each group.

	Monovalent booster (*n* = 54)	Bivalent booster (*n* = 24)	Omicron BA.5 recovered (*n* = 13)
Mean age (± SD)	53.98 (±3.78)	49.33 (±7.87)	25.35 (±6.95)
Gender (M/F)	28/26	17/7	5/8
Individuals with previous SARS‐CoV‐2 infection	49	21	—
Previous vaccination doses (monovalent ancestral vaccine)			
4 doses	0	11	0
3 doses	49	10	13
Individuals without previous SARS‐CoV‐2 infection (previous monovalent ancestral vaccination)	5 (3 doses)	3 (3 doses)	—
Infected before Omicron emergence	0	0	—
Infected during Omicron circulation	38	18	—
Median date of previous infection	2022‐03‐14 (±79)	2022‐3‐24 (±77)	2022‐9‐7 (±12)
Infection date unknown (serum Ab positive in NP ELISA without positive questionnaire)	11	3	
Median date (1 month) sample collection	2022‐9‐28 (±2)	2023‐2‐14 (±24)	2022‐10‐6 (±12)

### 3.1 Cross‐Neutralization Activities of Monovalent and Bivalent Booster Recipients and BA.5‐Recovered Patients Against Ancestral and Recent Omicron Variants

**FIGURE 1:**
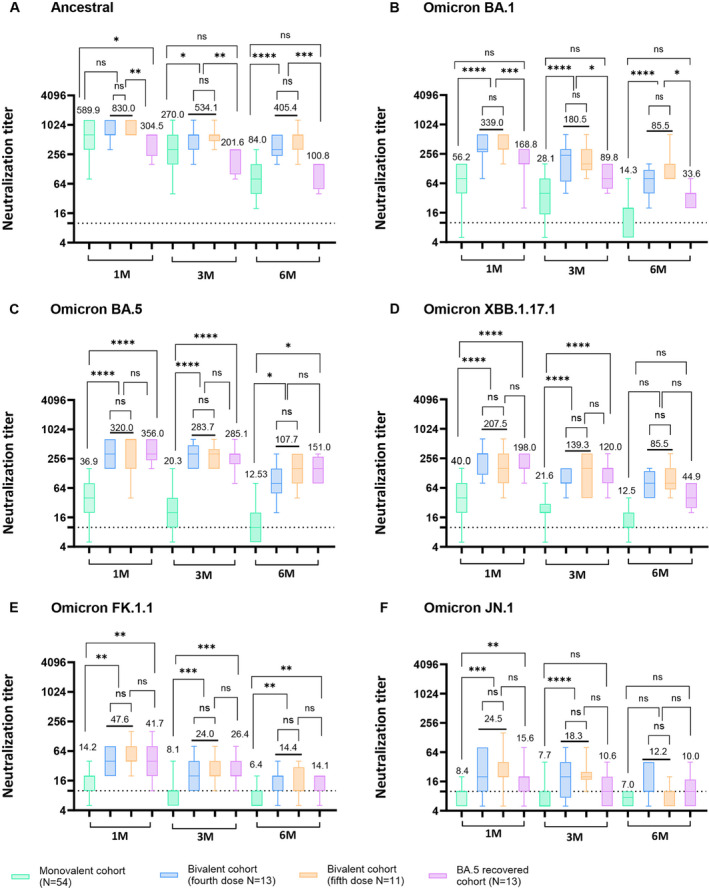
Longitudinal evaluation of serum neutralization against SARS‐CoV‐2 strains and variants after monovalent or bivalent vaccination or BA.5 breakthrough infection. (A–F) Serum samples serially collected from individuals vaccinated with monovalent or bivalent boosters and individuals recovered from BA.5 infections were assessed for cross‐neutralizing activity against (A) SARS‐CoV‐2 ancestral (WIV‐04; L), (B) Omicron BA.1, (C) Omicron BA.5, (D) Omicron XBB.1.17.1, (E) Omicron FK.1.1, and (F) Omicron JN.1 Dashed line indicates the first serum dilution (1:10) of the NAb assay. Statistical analysis was performed using Student's two‐tailed unpaired *t*‐tests. ns, not significant, *p < 0.05; **p < 0.01; ***p < 0.001; ****p < 0.0001. The neutralizing activity tests were repeated three times independently. Numbers are the computed geometric mean titers of sera at 1, 3, and 6 months.

Both monovalent (ancestral) and bivalent (ancestral and BA.4/BA.5) booster recipients exhibited robust serum NAb titers against the ancestral SARS‐CoV‐2 strain at 1 month (GMT 589.9 and GMT 830.0) and 3 months (GMT 270.0 and GMT 534.1) post‐booster vaccinations. Similarly, BA.5‐recovered patients showed detectable serum NAb titers against the ancestral strain 1 month (GMT 304.5) and 3 months (GMT 201.6) post‐BA.5 infections. Notably, at 6 months post‐booster vaccination, bivalent vaccine recipients demonstrated significantly higher NAb titers (GMT 405.4) compared to both monovalent recipients (GMT 84.0) and BA.5‐recovered HCWs (GMT 100.8) against the SARS‐CoV‐2 ancestral strain (Figure 1A; *p* < 0.0001 and *p* < 0.001). Interestingly though, the difference in GMT NAb titers against all tested SARS‐CoV‐2 strain (ancestral, Omicron BA.1, BA.5. XBB.1.17.1, FK.1.1, and JN.1) in the bivalent booster, whether given as a fifth dose or a fourth dose, was not statistically significant (Figure 1A–F).

The NAb titers in the sera of bivalent booster recipients were significantly higher than those in the sera of monovalent booster recipients against the BA.1 sublineage at 1 month post‐booster vaccinations (GMT 339.0 vs. GMT 56.2, *p* < 0.0001). Moreover, BA.5‐recovered individuals exhibited higher NAb titers against BA.1 (GMT 168.8) compared to monovalent booster recipients (GMT 56.2) at 1 month post‐infection (*p* < 0.001). This pattern continued up to 3 and 6 months post‐booster or SARS‐CoV‐2 BA.5 infection (Figure 1B).

For the Omicron BA.5 sublineage, the GMT of NAb in bivalent booster recipients (GMT 320.0) and BA.5‐recovered patients (GMT 356.0) were significantly higher than that in monovalent booster recipients (GMT 36.9) (Figure 1C; *p* < 0.0001). Notably, serum NAb levels against the ancestral strain (GMT 830.0) were significantly elevated in individuals who received bivalent booster vaccinations compared to their corresponding NAb titer against Omicron BA.1 (GMT 339.0) and Omicron BA.5 (GMT 320.0) (Figure S1C).

For the XBB.1.17.1 sublineage, the GMT of NAb in bivalent booster recipients (GMT 207.5) and BA.5‐recovered individuals (GMT 198.0) was significantly higher than that in monovalent booster recipients (GMT 40.0) at 1 month post‐vaccination or SARS‐CoV‐2 BA.5 infections (Figure 1D; *p* < 0.0001), indicating weaker protection of monovalent booster recipients against the XBB.1.17.1 sublineage than of bivalent booster recipients or SARS‐CoV‐2 BA.5‐recovered patients.

For the FK.1.1 sublineage bivalent booster recipients and BA.5‐recovered individuals, the GMT of NAb exceeded 40 units 1 month post‐vaccination, suggesting a protective effect against the FK.1.1 sublineage. Nonetheless, the GMT NAb levels of these groups declined rapidly at 3 and 6 months (Figure 1E).

Serum neutralizing activity against JN.1 sublineage was the lowest across all tested groups. Bivalent booster recipients exhibited a GMT above 20 units at 1 month post‐vaccination and remained detectable until 3 months post‐vaccination. For the other cohorts, the same trend of serum NAb against FK.1.1 sublineage was observed (Figure 1F).

### 3.2 Comparison of Cross‐Neutralizing Activity in Individuals of Each Group Without Prior SARS‐CoV‐2 Infection

To compare the pure vaccination effect of monovalent and bivalent booster vaccination, we selected individuals with no previous SARS‐CoV‐2 infection confirmed by both the questionnaire on their SARS‐CoV‐2 diagnosis history and ELISA test against the SARS‐CoV‐2 NP protein (Figure S1B). Five of the 54 monovalent booster recipients and three of the 24 bivalent booster recipients had no previous SARS‐CoV‐2 infection.

**FIGURE 2:**
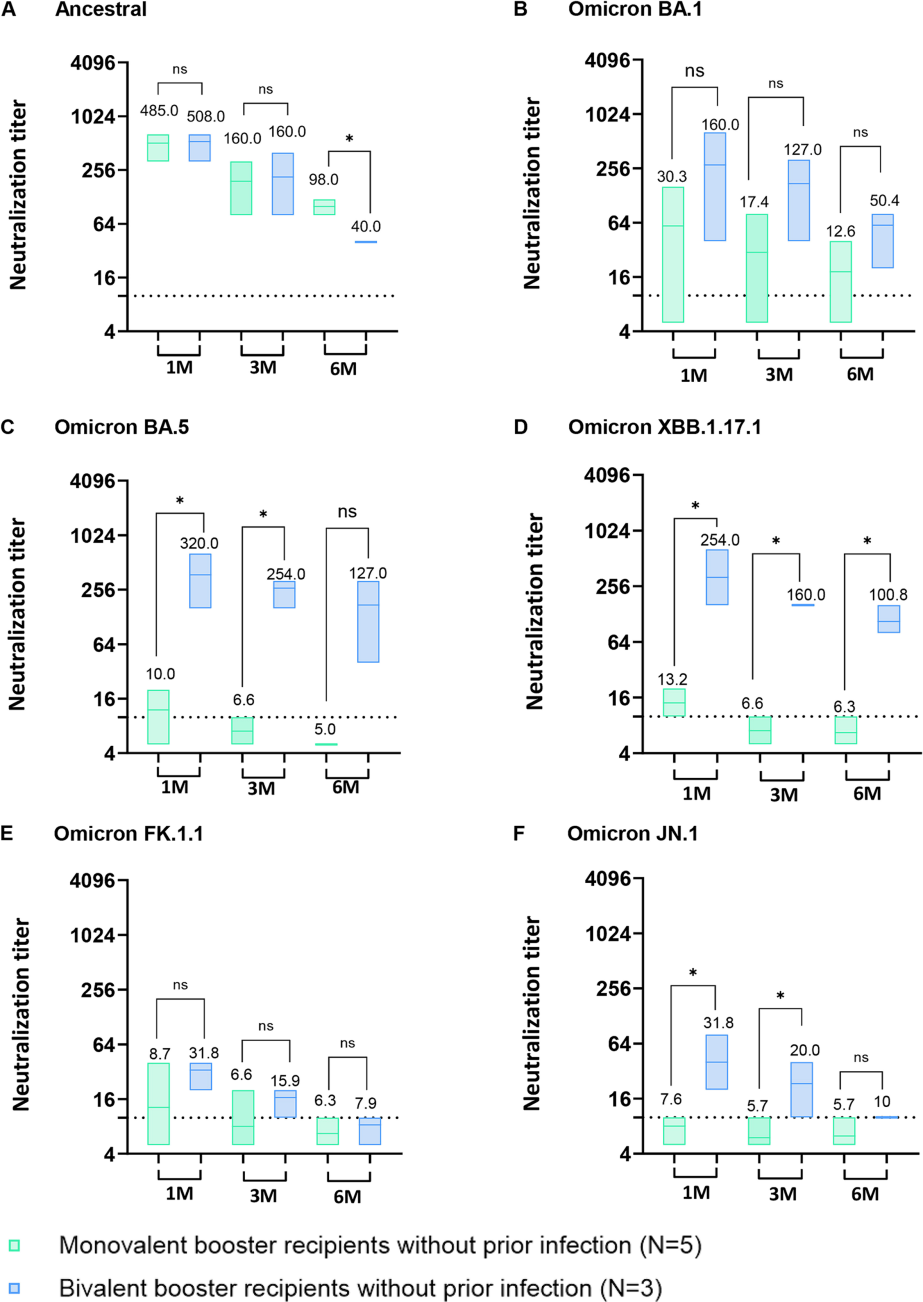
Serum‐neutralizing antibody titers from individuals without prior SARS‐CoV‐2 infection. (A–F) Serum samples serially collected from monovalent and bivalent booster recipients without prior SARS‐CoV‐2 infection were assessed for cross‐neutralizing activity against (A) SARS‐CoV‐2 ancestral (WIV‐04; L), (B) Omicron BA.1, (C) Omicron BA.5, (D) Omicron XBB.1.17.1, (E) Omicron FK.1.1, and (F) Omicron JN.1 Dashed line indicates the first serum dilution (1:10) of the NAb assay. Statistical analysis was performed using Student's two‐tailed unpaired *t*‐tests. ns, not significant, *p < 0.05; **p < 0.01; ***p < 0.001; ****p < 0.0001. The neutralizing activity tests were repeated three times independently. Numbers are the computed geometric mean titers of sera at 1, 3, and 6 months.

Among participants without prior SARS‐CoV‐2 infection before study enrollment, GMT NAb of monovalent booster recipients against the ancestral SARS‐CoV‐2 was not significantly different from that of bivalent recipients at 1 and 3 months, but the level of NAb of monovalent booster recipients (GMT 98.0) was significantly higher than that of bivalent recipients (GMT 40.0) at 6 months post‐vaccination (*p* < 0.05; Figure 2A). However, GMT of NAb against Omicron BA.1 was relatively high in bivalent recipients compared with those of monovalent recipients (Figure 2B).

Bivalent booster recipients without prior infection exhibited significantly elevated NAb against Omicron BA.5 at 1 and 3 months post‐vaccination compared to monovalent recipients without prior infection (Figure 2C; *p* < 0.05). Bivalent booster recipients without prior SARS‐CoV‐2 infection had significantly higher NAb activity against Omicron XBB.1.17.1 at 1 up to 6 months post‐vaccination than to monovalent booster recipients (*p* < 0.05), but there was no significant difference against Omicron FK.1.1 (Figure 2D,E). Serum NAbs against Omicron JN.1 sublineage of bivalent booster recipients without prior infection were significantly higher at 1 month (GMT 31.8) and 3 months (GMT 20.0) compared to monovalent booster recipients without prior infection (GMT 7.6 and GMT 5.7) (Figure 2F; *p* < 0.05). GMT NAbs of monovalent vaccinees without prior infection against Omicron BA.5, XBB.1.17.1, FK.1.1, and JN.1 were observed to be below the detection level at 6 months post‐vaccination (Figure 2C–F).

### 3.3 Comparison of Cross‐Neutralizing Activity Between Individuals With Previous SARS‐CoV‐2 Infection and Those Without Previous Infection

**FIGURE 3:**
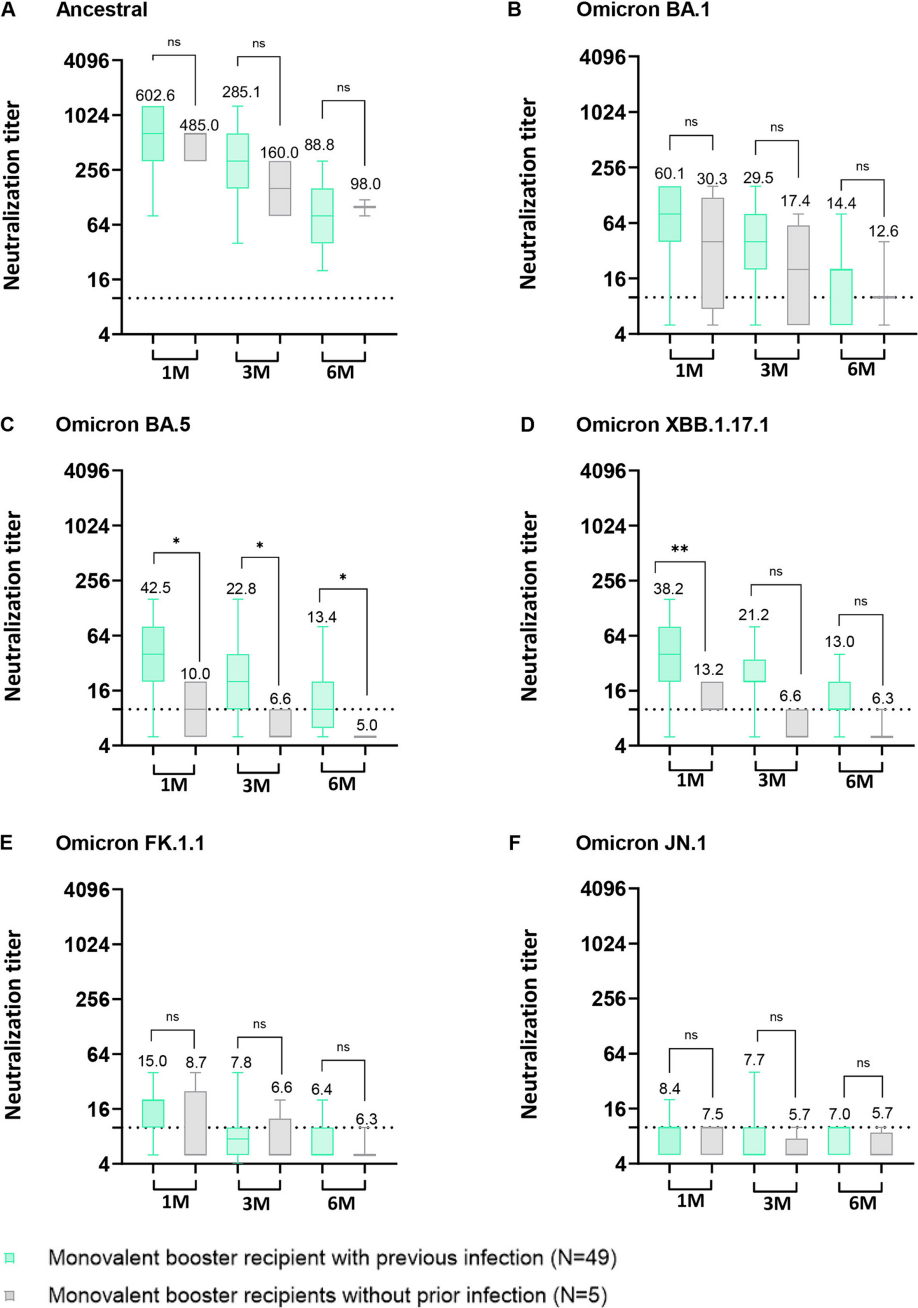
Comparison of serum neutralizing antibody titers of individuals with prior SARS‐CoV‐2 infection and without previous SARS‐CoV‐2 infection prior to monovalent booster vaccination. (A–F) Serum samples serially collected from monovalent booster recipients with and without prior SARS‐CoV‐2 infection were assessed for cross‐neutralizing activity against (A) SARS‐CoV‐2 ancestral (WIV‐04; L), (B) Omicron BA.1, (C) Omicron BA.5, (D) Omicron XBB.1.17.1, (E) Omicron FK.1.1, and (F) Omicron JN.1 Dashed line indicates the first serum dilution (1:10) of the NAb assay. Statistical analysis was performed using Student's two‐tailed unpaired *t*‐tests. ns, not significant, *p < 0.05; **p < 0.01; ***p < 0.001; ****p < 0.0001. The neutralizing activity tests were repeated three times independently. Numbers are the computed geometric mean titers of sera at 1, 3, and 6 months.

To evaluate the differential impact of vaccination between individuals with prior SARS‐CoV‐2 infection and those without prior infection, we segregated both monovalent and bivalent booster recipients into two distinct groups: those with a history of previous SARS‐CoV‐2 Omicron infection and those without. All of the previous SARS‐CoV‐2 infection occurred between February 2022 and December 2022, suggestive of Omicron infection. In the case of monovalent booster recipients, both groups exhibited high NAb titers against the ancestral SARS‐CoV‐2 strain until 6 months post‐vaccination (Figure 3A). In the neutralization test against the Omicron BA.5 sublineage, individuals who had previously been infected with Omicron and received a monovalent booster displayed significantly higher geometric mean titers (GMT42.5, GMT 22.8, and GMT 13.4) at 1–6 months post‐booster vaccination in comparison to those who had not been previously infected with Omicron (Figure 3C, *p* < 0.05). For BA.1, the group with prior Omicron infection displayed elevated GMT of NAbs though these differences did not reach statistical significance (Figure 3B). For Omicron XBB.1.17.1, individuals with prior Omicron infection who received a monovalent booster displayed significantly higher geometric mean titers at 1 month post‐vaccination compared to those without previous infection (Figure 3D; *p* < 0.01). In the serum neutralization assay against Omicron FK.1.1 and JN.1, both groups exhibited very low GMT of NAbs (Figure 3E,F).

**FIGURE 4:**
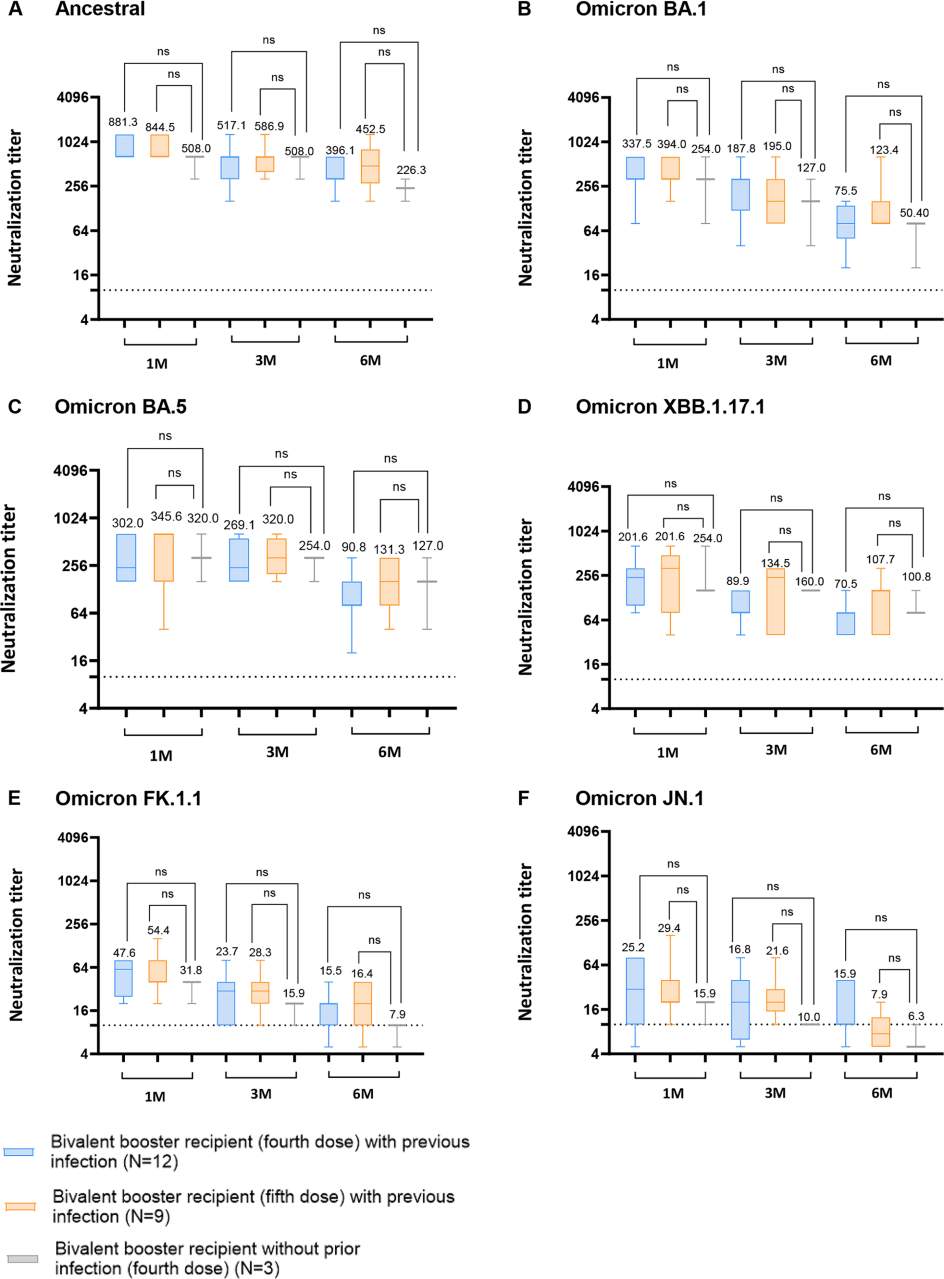
Comparison of serum neutralizing antibody titers of individuals with prior SARS‐CoV‐2 infection and without previous SARS‐CoV‐2 infection prior to bivalent booster vaccination. (A–F) Serum samples serially collected from bivalent booster recipients with or without prior SARS‐CoV‐2 infection were assessed for cross‐neutralizing activity against (A) SARS‐CoV‐2 ancestral (WIV‐04; L), (B) Omicron BA.1, (C) Omicron BA.5, (D) Omicron XBB.1.17.1, (E) Omicron FK.1.1, and (F) Omicron JN.1 Dashed line indicates the first serum dilution (1:10) of the NAb assay. Statistical analysis was performed using Student's two‐tailed unpaired *t*‐tests. ns, not significant, *p < 0.05; **p < 0.01; ***p < 0.001; ****p < 0.0001. The neutralizing activity tests were repeated three times independently. Numbers are the computed geometric mean titers of sera at 1, 3, and 6 months.

Among bivalent booster recipients, the group with prior Omicron infection displayed higher GMT of NAbs against the ancestral strain from 3 months to 6 months post‐vaccination than those of bivalent booster recipients without prior Omicron infection (Figure 4A). The same trend was observed between 1 and 3 months post‐vaccination against Omicron BA.1 (Figure 4B). However, when NAb titers were assessed against Omicron BA.5, XBB.1.17.1, FK.1.1, and JN.1, the differences observed between the two groups were not statistically significant (Figure 4C–F).

## 4 Discussion

The persistent expansion of the highly divergent Omicron variant into distinct sublineages, characterized by key mutations that augment their ability to evade preexisting population immunity induced by vaccination or prior infections, has raised significant concerns about the adequacy of the currently employed vaccine strain. Of particular concern is the growing evidence of antibody evasion and heightened infection rates associated with Omicron sublineages harboring additional mutations within the receptor‐binding domain (RBD) of the spike glycoprotein, such as R346T and K444T. These mutations, located outside the receptor‐binding motif (RBM) and within the epitope of Class III NAbs, have been shown to disrupt antibody recognition, as revealed by structural analyses [4, 17]. Consequently, there has been an alarming surge in Omicron reinfections and instances of vaccine escape [3].

In our study, we observed that individuals who received bivalent booster doses exhibited significantly enhanced neutralizing activity against various Omicron sublineages (BA.1, BA.5, XBB.1.17.1,FK.1.1, and JN.1) compared to those who received monovalent booster vaccine. Individuals with recent Omicron BA.5 infection also showed high serum neutralizing activity against Omicron BA.1 and Omicron XBB.1.17.1 sublineages. For the Omicron FK.1.1 sublineage, bivalent recipients and recent BA.5‐recovered individuals showed significantly higher serum neutralizing activity than monovalent recipients. Against the recent Omicron JN.1 sublineage, bivalent booster recipients regardless if it is given as a fourth dose or as a fifth dose had significantly higher NAb titers than monovalent booster (based on ancestral strain) recipients. These findings align with recent reports indicating that individuals who received bivalent booster shots mounted more robust NAb titers when compared to those who received a series of monovalent vaccines [3, 4]. However, it is noteworthy that the elicited neutralizing titers against XBB.1.17.1, FK.1.1, and JN.1 remained relatively modest in the bivalent booster recipients and were exceedingly low in the monovalent booster recipients.

Our results also demonstrate that recent SARS‐CoV‐2 Omicron‐variant infection prior to either monovalent or bivalent booster vaccination enhanced neutralizing activity and breadth of the antibody response against the ancestral strains BA.1 and BA.5, as well as recent Omicron sublineages (XBB.1.17.1, FK.1.1, and JN.1) compared to those individuals without prior SARS‐CoV‐2 Omicron infection. These results corroborate the previous findings that *hybrid immunity* induced by vaccination following recovery from SARS‐CoV‐2 natural infection substantially increases the potency of the antibody response [18, 19]. However, we acknowledge that the small sample size of uninfected participants in both the monovalent (five individuals) and (three individuals) booster groups is a limitation that could affect the statistical power and generalizability of our findings.

Interestingly, our results revealed that individuals recovered from recent Omicron BA.5 infection displayed comparable NAb titers against the ancestral SARS‐CoV‐2 strain compared to the monovalent recipients. Furthermore, against Omicron BA.1 and BA.5, these individuals exhibited similar serum NAb titers when compared to bivalent booster recipients. Notably, the geometric mean titer of NAbs against XBB.1.17.1 and FK.1.1 are higher in Omicron BA.5‐recovered patients compared to monovalent booster recipients. This is likely because all of the BA.5‐recovered patients had received three doses of the SARS‐CoV‐2 monovalent vaccine, and thus, their Omicron BA.5 breakthrough infections could have stimulated their antibody responses against pan‐SARS‐CoV‐2 variants [15]. These pan‐SARS‐CoV‐2 antibody responses following BA.5 virus infection can be attributed to various factors, including exposure to multiple viral antigens, epitope spreading, and somatic hypermutation of B cells. Furthermore, these responses are influenced by the adaptability of the immune system, its ability to recognize conserved viral regions across variants like the S2 fusion subunit [20], and the role of inflammation in enhancing the immune response. Although these antibodies offer broad recognition, their efficacy against different variants may exhibit variation.

Notably, the bivalent BA.4/BA.5 vaccine significantly enhanced NAb titers against the ancestral strain. However, NAb titers against Omicron BA.5 were comparatively lower, aligning with prior research [21] and potentially attributable to immune imprinting Subsequent boosting with updated SARS‐CoV‐2 vaccines may further prime individuals' immune responses to recognize epitopes shared by these strains, rather than exclusively targeting the novel epitopes of new variants. Furthermore, recent findings suggest that the elevated IgG4 levels induced by repeated mRNA vaccinations might function as an immune tolerance mechanism against the SARS‐CoV‐2 spike protein. This mechanism allows infection and replication to proceed unhindered while concurrently dampening innate antiviral responses. Consequently, this could result in an increased susceptibility to new Omicron sublineages. [22].

Our study highlights the urgent and persistent need for the development of targeted vaccines against Omicron sublineages. Despite progress in broadening neutralization with bivalent boosters, effectively neutralizing a subset of emerging Omicron subvariants remains a challenge. As the virus continues to evolve, it is imperative to continually adapt vaccine strategies to address these variants in order to safeguard public health.

In conclusion, our study highlights the potential benefits of the bivalent booster vaccine in enhancing NAb responses against emerging Omicron sublineages, including XBB 1.17.1, FK.1.1, and JN.1. In the general population, the previously administered bivalent booster vaccine appears to provide protective effects against these novel Omicron sublineages (XBB.1.17.1, FK.1.1, and JN.1). Furthermore, our findings suggest that recent breakthrough infections with Omicron sublineages could potentially offer some degree of cross‐protection against other emerging Omicron subvariants. Nevertheless, the relatively modest neutralization observed for specific Omicron sublineages and the rapid decline in antibody titers underscore the need for the development of tailored SARS‐CoV‐2 vaccines, especially for immunocompromised or high‐risk populations.

## Author Contributions

Conceptualization: H.W.J., S.M.K., R.R., and Y.K.C. Methodology, H.W.J., S.M.K., R.R., J.G., M.A.C., H.J., and J.H.C. Investigation: H.W.J., S.M.K., R.R., J.G., M.A.C., H.J., J.H.C., S.G.J., J.C.L., H.S.K., and J.H.K. Visualization: H.W.J., S.M.K., R.R. and Y.K.C. Funding acquisition: H.W.J. and Y.K.C. Writing–original draft: H.W.J., S.M.K., R.R., and Y.K.C. Writing–review and editing: H.W.J., S.M.K., R.R., and Y.K.C.

## Ethics Statement

This study was carried out in accordance with the study protocol approved by the Institutional Review Board of Chungbuk National University Hospital (IRB no. 2022‐04‐013, 2021‐02‐010). Informed consent was obtained from all individuals enrolled in this study. All SARS‐CoV‐2 handling experiments were carried out in a BSL3 facility at Chungbuk National University (approval number CBNUA‐1352‐20‐02).

## Conflicts of Interest

The authors declare no conflicts of interest.

## Supporting information


**Figure S1.** Schematic protocol of sera collection from enrolled individuals and NP ELISA results. (A) Schematic image of the sera from monovalent (*n* = 54), bivalent booster recipient as fourth dose (*n* = 13), bivalent booster recipient as fifth dose (*n* = 11), and BA.5 recovered patients (n = 13). Enrolled individuals were serially sampled at 1, 3, and 6 months after their booster dose or BA.5 infection. Created with biorender.com. (B) ELISA analysis using anti‐SARS‐CoV‐2 NP immunoglobulin G (IgG) antibodies was performed to assess prior exposure of the subjects to SARS‐CoV‐2. (C) Summary of NAb titers against (Ancestral, Omicron BA.1, and Omicron BA.5) comparison in bivalent booster recipients. Statistical analysis was performed using the Student’s two‐tailed unpaired t‐tests. ns, not significant, *p < 0.05; **p < 0.01; ***p < 0.001; ****p < 0.0001. The neutralizing activity tests were repeated three times independently. Numbers are the computed geometric mean titers of sera at 1 month post‐vaccination

## Data Availability

The data that support the findings of this study are available from the corresponding author upon reasonable request.
