# Supplementary figures and images for: Enhancing Omicron Sublineage Neutralization: Insights From Bivalent and Monovalent COVID‐19 Booster Vaccines and Recent SARS‐CoV‐2 Omicron Variant Infections
Source: Influenza Other Respir Viruses. 2024 Oct 8;18(10):e70000. doi: 10.1111/irv.70000 (PMC11459205; doi:10.1111/irv.70000)

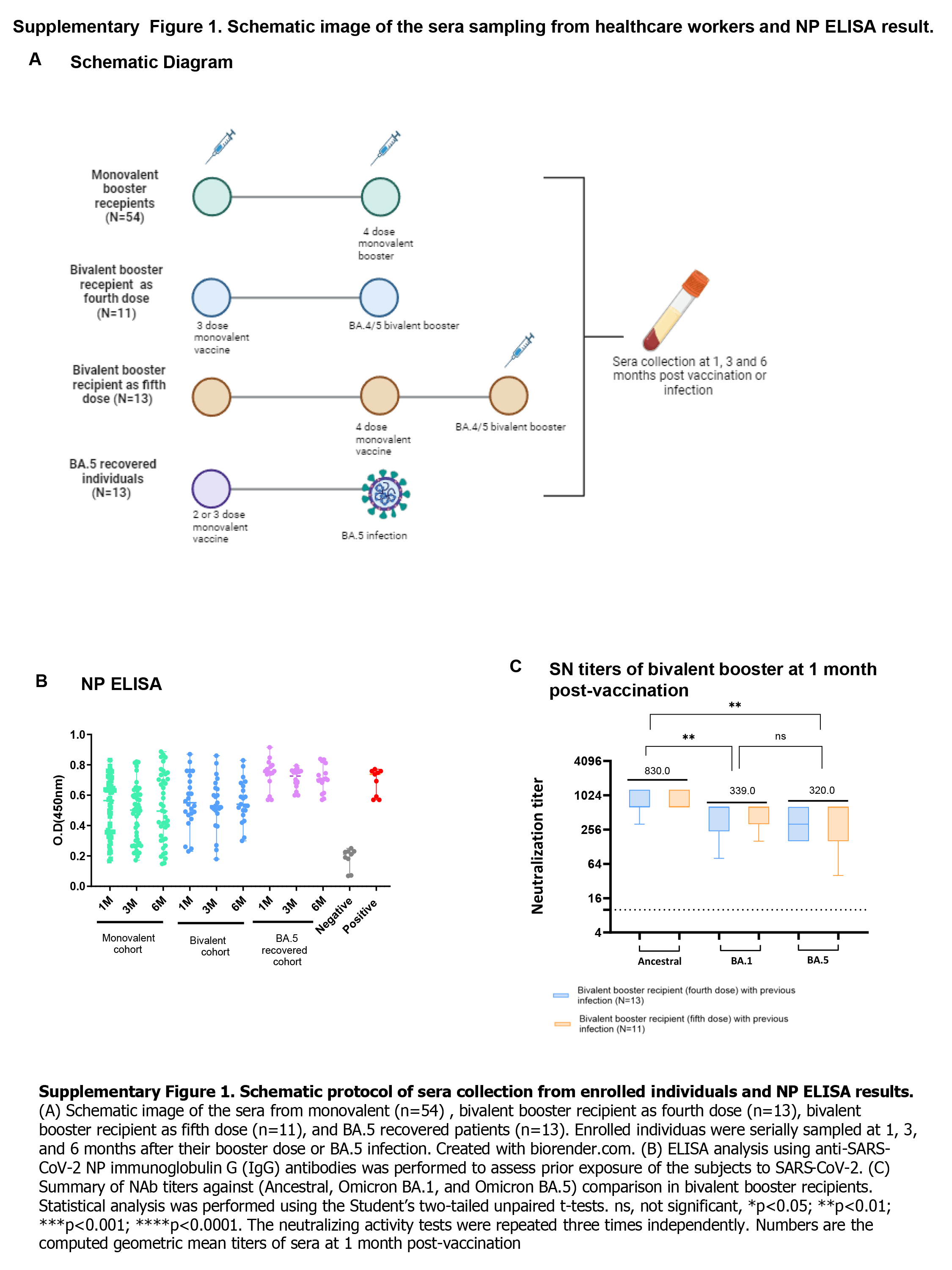

Supplement: Supplementary file 1 — Figure S1. Schematic protocol of sera collection from enrolled individuals and NP ELISA results. (A) Schematic image of the sera from monovalent (n = 54), bivalent booster recipient as fourth dose (n = 13), bivalent booster recipient as fifth dose (n = 11), and BA.5 recovered patients (n = 13). Enrolled individuals were serially sampled at 1, 3, and 6 months after their booster dose or BA.5 infection. Created with biorender.com. (B) ELISA analysis using anti‐SARS‐CoV‐2 NP immunoglobulin G (IgG) antibodies was performed to assess prior exposure of the subjects to SARS‐CoV‐2. (C) Summary of NAb titers against (Ancestral, Omicron BA.1, and Omicron BA.5) comparison in bivalent booster recipients. Statistical analysis was performed using the Student’s two‐tailed unpaired t‐tests. ns, not significant, *p < 0.05; **p < 0.01; ***p < 0.001; ****p < 0.0001. The neutralizing activity tests were repeated three times independently. Numbers are the computed geometric mean titers of sera at 1 month post‐vaccination [file IRV-18-e70000-s001.tif]
